# Evaluation of left atrial deformation to predict left atrial stasis in patients with non-valvular atrial fibrillation – a pilot-study

**DOI:** 10.1186/1476-7120-11-44

**Published:** 2013-12-19

**Authors:** Rui Providência, Ana Faustino, Maria João Ferreira, Lino Gonçalves, Joana Trigo, Ana Botelho, Sérgio Barra, Serge Boveda

**Affiliations:** 1 Serviço de Cardiologia, Centro Hospitalar e Universitário de Coimbra, Praceta Prof. Mota Pinto, Coimbra 3000-075, Portugal; 2 Faculty of Medicine, University of Coimbra, Coimbra, Portugal; 3 Papworth Hospital NHS Foundation Trust, Cambridge, United Kingdom; 4 Département de Rythmologie, Clinique Pasteur, Toulouse, France

**Keywords:** Speckle tracking, Strain, Strain rate, Left atrium, Left atrial appendage, Non-valvular atrial fibrillation, Stasis, Thrombus, Sludge

## Abstract

**Background:**

Speckle tracking-derived strain and strain rate are recently available parameters to assess left atrial (LA) deformation. We hypothesized that such new parameters could be of interest to evaluate the risk of LA stasis among patients with atrial fibrillation (AF).

**Methods:**

Single-centre study enrolling all patients with non-valvular AF lasting longer than 48 hours for whom no therapeutic anticoagulation was given in the preceding 3 weeks and who were assessed through transesophageal and transthoracic echocardiogram during a 6 month time interval. LA deformation was assessed by transthoracic echocardiogram through speckle tracking analysis, whereas LA stasis parameters were sought on transesophageal echocardiogram.

**Results:**

Among the 82 assessed patients, LA appendage thrombi or sludge were found in 16 (19.5%). A moderate positive correlation was found between peak positive strain rate and maximum emptying velocity (r = 0.589; *P* <0.001) and peak positive strain rate and maximum filling velocity of the LA appendage (r = 0.651; *P* <0.001). Peak negative strain rate was also found to be associated with both maximum emptying velocity (r = -0.513; *P* <0.001) and maximum filling velocity of the LAA (r = -0.552; *P* <0.001). AF duration, peak negative strain rate and time-to-peak positive strain were independent predictors of LAA thrombi or sludge on multivariate analysis logistic regression. The area under the curve for the estimated probabilities using the obtained logistic regression model was 0.89 (95%CI 0.81-0.96; *P* <0.001).

**Conclusion:**

Our findings suggest that LA mechanical dysfunction assessed through speckle tracking may be of interest to predict LA stasis in the setting of AF.

## Background

Non-valvular atrial fibrillation (AF) is associated with stroke and peripheral embolism of cardiac origin [1] and over 90% of thrombi are thought to originate in the left atrial appendage (LAA) [2].

Transesophageal echocardiogram is the gold-standard for the detection of thrombi in the LAA [3] and its use before cardioversion and catheter ablation of AF has grown in recent years [4]. This is a semi-invasive technique that despite a very low incidence of complications carries risks over transthoracic imaging [5].

Transthoracic echocardiography is now a highly versatile technique that provides solid structural and functional information about the atria. Its potential role in the risk stratification of AF and prediction of left atrial stasis has been overlooked [3]. Speckle tracking is an imaging technique that provides accurate and angle-independent information regarding left atrial deformation and motion [6]. Echocardiographic parameters assessing structure, like left atrial size, are known to impact on the presence of left atrial stasis (thrombi or sludge, dense spontaneous echocardiographic contrast and low flow velocities in the LAA) [7].

We hypothesized that speckle tracking derived strain and strain rate could be of interest to evaluate the risk of LA stasis among patients with atrial fibrillation (AF).

## Methods

### Patient selection

All patients during a 6 month time interval undergoing transthoracic and transesophageal echocardiogram were assessed for the presence of criteria allowing admission into the study. Patients with an AF episode lasting for longer than 48 hours and without effective anticoagulation in the preceding 3 weeks were considered eligible for the study except if any of the following exclusion criteria were met: lack of adequate endocardial border definition of the left atrium, presence of prosthetic heart valve or previous valve repair, significant aortic or mitral valve disease (any degree of aortic/mitral valve stenosis or mitral/aortic regurgitation > II/IV) and previous closure of the LAA.

This study was conducted with the approval of our Institution’s Ethics Committee, Comissão de Ética da Faculdade de Medicina da Universidade de Coimbra. All subjects provided their informed consent to undergo the necessary investigations and to allow the usage of their data for research purposes, preserving their anonymity.

Preliminary transthoracic echocardiography was performed using standard views (GE Vivid 7 echocardiograph with a M4S probe). The frame rate was set > 60 frames per second. Since all patients were in AF at the time of procedure, all measurements were obtained from an average of 3 cycles. Left atrium volume was measured using the bi-plane area length method. Left ventricle ejection fraction was calculated with the Simpson method. The ratio between indexed left atrial volume and left ventricle ejection fraction, which has shown to be highly accurate at excluding the presence of an LAA thrombus in patients with AF who are candidates for AF catheter ablation or cardioversion [7], was calculated.

Pulsed-wave Doppler at the tips of the mitral valve was used for measuring early diastolic filling velocity (E). The early diastolic tissue velocity (E’) was measured with tissue Doppler imaging of the lateral mitral annulus. E/E’ ratio was calculated. Mitral regurgitation was semi-quantitatively assessed by color Doppler across mitral valve and graded as none/trace (0), mild (I/IV) and moderate (II/IV). Individuals with moderately severe (III/IV) and severe (IV/IV) mitral regurgitation were excluded from analysis.

Global longitudinal strain and strain rate of the left ventricle were assessed as previously described by other authors [8,9].

### Assessment of left atrial deformation by transthoracic echocardiogram

Transthoracic images were processed for assessing left atrial deformation through speckle tracking imaging using the GE Health Care EchoPac Dimension software, PC version 108.1.4 (featuring a software for speckle-tracking of the left ventricle) by two cardiologists who were blinded for the transesophageal echocardiogram results and clinical information of the patients.

**Figure 1:**
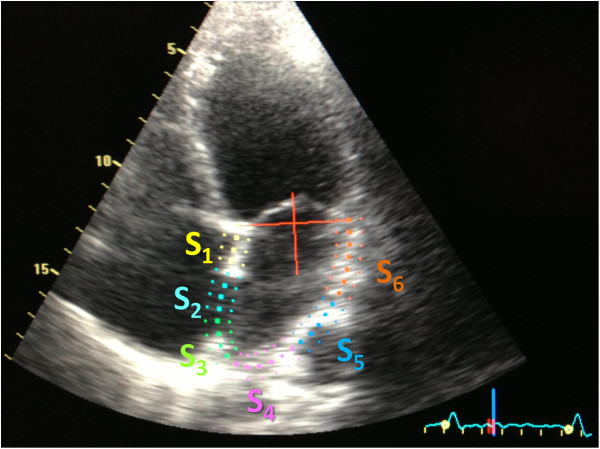
Assessed segments in the left atrial region of interest.

**Figure 2:**
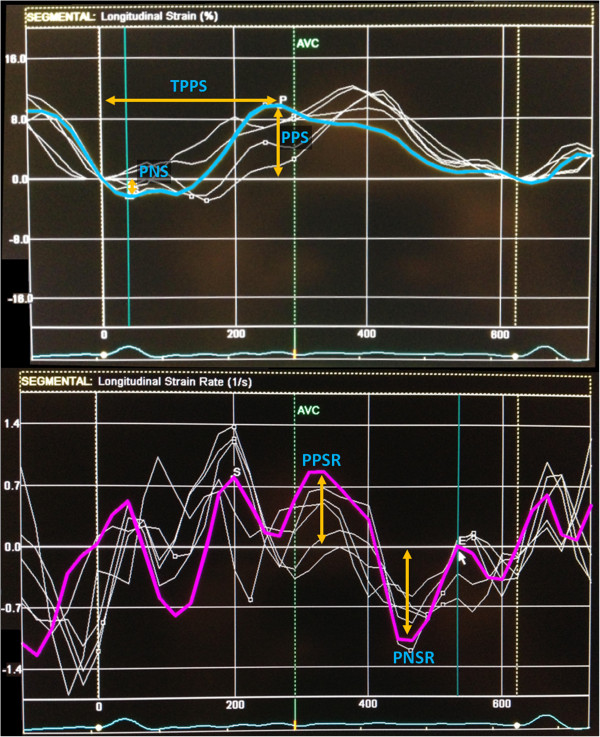
**Left atrial longitudinal deformation and dyssynchrony measurements.** Legend: Peak negative strain (PNS) and peak positive strain (PPS) were measured of each segment from baseline to the peak negative or peak positive value of longitudinal strain, respectively; Peak positive strain rate (PPSR) and peak negative strain rate (PNSR) of each segment were measured from baseline to the peak positive or peak negative value of longitudinal strain rate, respectively. The average of all 6 segments during 3 cardiac cycles was calculated. Time to peak positive strain (TPPS) was the time interval measured from the beginning of the cardiac cycle to the timing of peak positive strain; the standard deviation of all 6 segments during 3 cardiac cycles was calculated.

The left atrium endocardial surface was manually traced using a point-and-click approach in apical four-chamber view, which allowed the automatic definition of a region of interest. This was manually adjusted, if necessary, to better suit the atrium anatomy. The cardiac cycle was demarcated by indicating QRS onset. The region of interest was divided into 6 segments by the software (Figure 1) and the resulting tracking quality for each segment was scored as either acceptable or non-acceptable. Segments classified as “non-acceptable” were rejected by the software and excluded from the analysis. In subjects with adequate image quality, a total of 6 segments were analyzed. Longitudinal strain and strain rate curves for each segment were analyzed and the following parameters collected (Figure 2): peak positive and peak negative strain, peak positive and peak negative strain rate and time to peak-positive strain. Peak-to-peak strain and strain rate were also calculated (i.e., peak positive – peak negative strain and peak positive – peak negative strain rate). These parameters referred to data from the whole cardiac cycle based on the assumption that since the left atrium was fibrillating during the entire cardiac cycle duration, there was no strong rationale for its division in different phases of atrial function (just like there would be no sense in assessing the different phases of ventricular function in a fibrillating ventricle). An average of the 6 segments in three consecutive heart cycles was estimated, except for time-to-peak positive strain, where standard deviation was calculated for three cycles also.

### Assessment of left atrial stasis by transesophageal echocardiogram

Transesophageal echocardiogram was performed without anaesthesia or sedation using a 6 T phased array multiplane transesophageal probe (frequency 7.0 MHz). The left atrium and LAA were imaged in different tomographic planes to detect the presence of LAA thrombus and spontaneous echocardiographic contrast, which was graded according to the classification (1 to 4+) proposed by Fatkin et al. [10]. When dense spontaneous echocardiographic contrast (grade 3+ or 4+) was present and organized into a dynamic and gelatinous, but not solid or well-formed, echodensity present throughout the cardiac cycle, sludge was reported [11].

LAA flow velocities were assessed with a pulsed Doppler sample placed 1 cm from the entry of the LAA into the body of the left atrium. Emptying and filling velocities were estimated from an average of five well-defined emptying and filling waves.

Patients were divided into 2 groups, according to the type of findings on transesophageal echocardiogram: group I, with LAA thrombus or sludge and group II, without any of these changes.

### Statistical analysis

Comparisons were performed between the two patient groups. Chi-square was used for nominal variables and Student’s t-test was used for comparison of continuous variables, where appropriate; the Levene’s test was used in order to check the homogeneity of variance; equivalent non-parametric tests were used when Kolmogorov-Smirnov was in favor of absence of normal distribution. Pearson’s r correlation coefficient was used for quantifying the degree of association between two quantitative variables. Results with p < 0.05 were regarded as significant.

Univariate analysis was performed using the chi-square test. Predictors from univariate analysis were used for obtaining logistic regression models (using the backward stepwise method likelihood ratio; probability for stepwise = 0.10) that could predict the presence of left atrial thrombi or sludge. Continuous variables which statistically differed between group I and II (or presented a non-significant trend: P < 0.1) were converted into qualitative variables and then used in the logistic regression analysis. Cut-off values were obtained through receiver operating characteristic (ROC) curves which allowed us to define the optimal cutoff point, which was the value combining the higher value of specificity plus sensitivity (Youden index). The Hosmer-Lemeshow summary statistic was used to assess the goodness-of-fit of the models. The coefficients from the obtained model (beta values from the incorporated variables and constant) were used for estimating the probability of event in each patient. Then, the discriminative capability of the obtained probabilities was also assessed through a ROC curve.

PASW Statistics version 18.0 was used for descriptive and inferential statistical analysis.

Inter-observer variability was assessed using Bland-Altman plots with data from the first 7 included patients in the study (average for each of the 6 segments shown), that were separately assessed by the two operators. MedCalc for Windows version 9.2.0.1 was used.

## Results

During the pre-specified inclusion period, 133 patients were assessed during an AF episode of longer than 48 hour duration and with no effective anticoagulation in the preceding 3 weeks. Of these, the following were excluded from analysis due to the presence of exclusion criteria: inappropriate endocardial border definition of the left atrium (n = 11), previous closure of the LAA (n = 3), valve repair or presence of prosthetic heart valve (n = 10) and significant valvular disease (n = 27).

Out of the remaining 82 patients, most were male (65.9%) and the average CHADS_2_ and CHA_2_DS_2_-VASc scores were 2.0 ± 1.1 and 3.2 ± 1.6, respectively. Information on demographics, clinical data and anti-thrombotic medication are provided on Table 1.

**Table 1:** Clinical data of the study sample

	**Overall**	**Group 1**	**Group 2**	**p**
	**(n = 82)**	**(n = 16)**	**(n = 66)**	
		**LAAT or sludge**	**No changes**	
**Demographics**				
Age	68.1 ± 10.6	70.7 ± 8.0	67.4 ± 11.1	0.516
Female gender	34.1% (28)	25% (4)	36.4% (24)	0.390
Body Mass Index (Kg/m^2^)	28.3 ± 4.1	26.6 ± 2.8	28.7 ± 4.3	0.100
**Clinical data**				
AF episode duration > 1 month	61.0% (50)	93.8% (15)	53.0% (35)	0.003
CHADS_2_	2.0 ± 1.1	2.3 ± 1.4	1.9 ± 1.1	0.418
CHA_2_DS_2_-VASc	3.2 ± 1.6	3.7 ± 1.6	3.1 ± 1.6	0.247
Congestive heart failure	43.9% (36)	50% (8)	42.4% (28)	0.584
Hypertension	80.5% (66)	93.8% (15)	77.3% (51)	0.136
Diabetes mellitus	18.3% (15)	31.3% (5)	15.2% (10)	0.135
Previous stroke or TIA	13.4% (11)	18.8% (3)	12.1% (8)	0.485
Vascular disease	15.9% (13)	25.0% (4)	13.6% (9)	0.264
**Anti-thrombotic medication**				
Aspirin	22.8% (18)	37.5% (6)	19.0% (12)	0.116
Clopidogrel	12.7% (10)	25.0% (4)	9.5% (6)	0.096
Warfarin	29.1% (23)	37.5% (6)	27.0% (17)	0.408
INR in patients treated with warfarin	2.4 ± 1.0	2.8 ± 1.6	2.2 ± 0.6	0.394
Dabigatran or Rivaroxaban*	15.2% (12)	18.8% (3)	14.3% (9)	0.657
Enoxaparin	35.4% (29)	25.0% (4)	37.9% (25)	0.334
Enoxaparin dosage (mg/Kg bid)	0.9 ± 0.2	1.0 ± 0	0.9 ± 0.2	0.288

Legend: LAAT – left atrial appendage thrombus; AF – atrial fibrillation; TIA – transient ischemic attack; INR – international normalized ratio.

*All patients were treated with dabigatran 110 mg bid except for two: one patient with rivaroxaban 20 mg id and another one with dabigatran 150 mg bid in group 2.

### Echocardiographic findings

Thrombus or sludge in the left atrium or LAA was found in 16 patients (19.5%) (group I). No differences were found for baseline variables when comparing these patients with the remaining (group II), except for the estimated AF duration, which was more often longer than 1 month in group I.

As regards standard transthoracic echocardiographic parameters, no differences were found between the two groups (Table 2). Also, no significant differences were found regarding left ventricular longitudinal strain and strain rate. On transesophageal echocardiogram lower flow velocities in the LAA and a higher prevalence of dense spontaneous echocardiographic contrast were observed in group I.

**Table 2:** Echocardiographic data of the study sample

	**Overall**	**Group 1**	**Group 2**	**P**
	**(n = 82)**	**(n = 16)**	**(n = 66)**	
		**LAAT or sludge**	**No changes**	
**Standard transthoracic echocardiogram data**				
Indexed LA volume (mL/m^2^)	47.0 ± 13.1	52.3 ± 13.2	45.8 ± 12.9	0.072
LVEF (%)	51.2 ± 12.7	49.3 ± 15.6	51.7 ± 12.0	0.734
Indexed LA volume / LVEF (mL/m^2^/%)	1.01 ± 0.55	1.24 ± 0.74	0.96 ± 0.49	0.153
Mitral regurgitation II/IV	18.3% (15)	12.5% (2)	19.7% (13)	0.504
E (cm/s)	97.8 ± 22.7	99.8 ± 20.0	97.2 ± 23.5	0.695
E’ (cm/s)	11.4 ± 2.4	11.6 ± 2.5	10.5 ± 1.9	0.113
E/E’ ratio	9.0 ± 3.2	9.8 ± 2.6	8.8 ± 3.3	0.268
**Speckle tracking derived parameters**				
Av. peak positive LA strain (%)	11.6 ± 5.2	10.9 ± 3.5	11.8 ± 5.5	0.433
Av. peak negative LA strain (%)	-4.0 ± 2.2	-3.5 ± 2.4	-4.1 ± 2.1	0.122
Av. peak-to-peak LA strain (%)	15.5 ± 5.0	14.4 ± 3.6	15.8 ± 5.3	0.312
Av. peak positive LA strain rate (s^-1^)	1.20 ± 0.32	1.00 ± 0.27	1.25 ± 0.32	0.003
Av. peak negative LA strain rate (s^-1^)	-1.37 ± 0.42	-1.07 ± 0.22	-1.45 ± 0.42	<0.001
Av. peak-to-peak LA strain rate (s^-1^)	2.6 ± 0.7	2.1 ± 0.4	2.7 ± 0.7	<0.001
SD time-to-peak positive LA strain (ms)	97.2 ± 47.1	110.4 ± 37.4	94.0 ± 48.9	0.097
LV global longitudinal systolic strain (%)	-12.6 ± 5.2	-12.3 ± 2.9	-12.6 ± 5.6	0.853
LV global longitudinal systolic strain rate (s^-1^)	-0.84 ± 0.22	-0.82 ± 0.17	-0.85 ± 0.23	0.617
**Transesophageal echocardiogram data**				
LAA maximum emptying flow velocity (cm/s)	26.8 ± 11.9	16.3 ± 4.2	29.2 ± 11.8	<0.001
LAA maximum filling flow velocity (cm/s)	33.8 ± 14.2	23.2 ± 8.4	36.2 ± 14.2	0.001
Dense spontaneous auto-contrast (≥ III/IV)	32.9% (27)	100% (16)	16.7% (11)	<0.001

Legend: LAAT – left atrial appendage thrombus; LA – left atrial; LV – left ventricle; LVEF – left ventricle ejection fraction; LAA – left atrial appendage; E – early diastolic filling velocity; E’ - early diastolic tissue velocity in the lateral mitral annulus; Av. – average; SD – standard deviation.

### LA deformation in patients with LA thrombus or sludge

Adequate tracking of all 6 segments was possible in most patients (n = 58; 70.7%). The segments which were more frequently excluded from analysis were Seg_6_ (in 18 patients; 22.0%) and Seg_1_1 (in 7 patients; 8.5%). In all the remaining segments, a satisfactory definition of the endocardial borders and tracking was possible (97.6% Seg_2_ 2; 93.9% Seg_3_; 96.3% Seg_4_; 96.3% Seg_5_).

**Figure 3:**
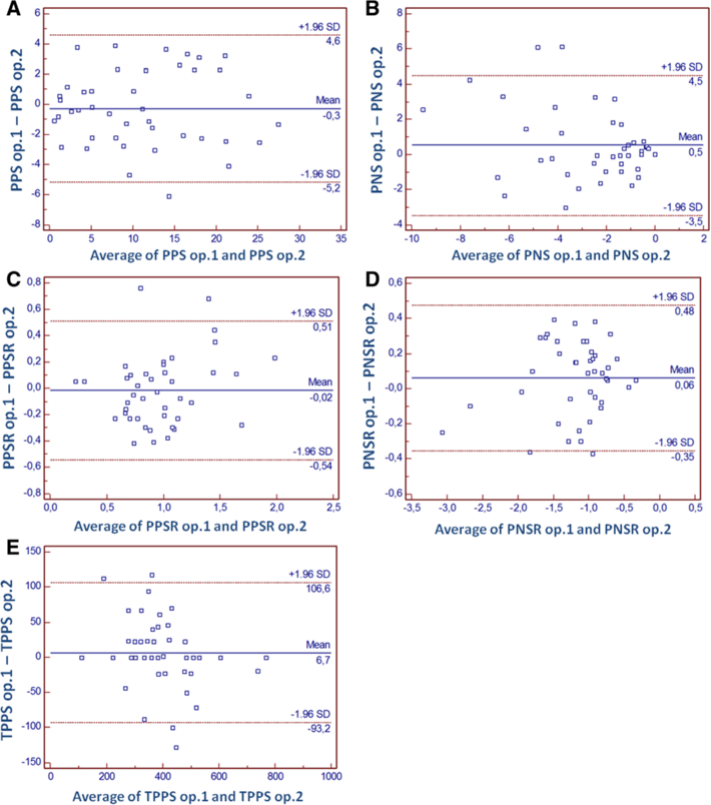
**Results of Bland-Altman analysis for interobserver variability regarding left atrial deformation.** Legend: peak positive strain (PPS – **A**.), peak negative strain (PNS – **B**.), peak positive strain rate (PPSR – **C**.), peak negative strain rate (PNSR – **D**.) and time-to-peak systolic strain (TPPS – **E**.).

Bland-Altman analysis for inter-observer variability of speckle-tracking derived data is shown in Figure 3. Small differences were observed overall for PPS (-0.3%; 95%CI -5.2; 4.6), PNS (0.5%; 95%CI -3.5; 4.5), PPSR (-0.02 s^-1^; 95%CI -0.54; 0.51), PNSR (0.06 s^-1^; 95%CI -0.35; 0.48) and TPPS (6.7 ms; 95%CI -93.2; 106.6).

Lower values of peak positive and peak negative strain rate, as well as a lower peak-to-peak strain rate, were observed in group I patients. Patients with sludge or thrombi had a trend for higher indexed left atrial volume and left atrial dyssynchrony, as assessed through the standard deviation of time to peak positive strain (Table 3).

**Table 3:** Univariate and multivariate analysis predictors of left atrial thrombus or sludge

	**Univariate analysis**			**Multivariate analysis**				
	**OR**	**95%CI**	**P**	**OR**	**95%CI**	**P**	**Wald**	**Hosmer-Lemshow**
BMI ≥ 26.9 kg/m^2^	0.3	0.1-1.0	0.049	-	-	-	-	
AF episode duration ≥ 1 month	13.3	1.7-106.5	0.003	13.3	1.5-119.6	0.021	5.3	
Indexed LAV ≥ 45.2 mL/m^2^	3.4	1.0-11.6	0.044	-	-	-	-	
Av. peak positive strain rate ≤ 1.01 (s^-1^)	6.3	1.9-20.9	0.001	-	-	-	-	χ^2^ = 1.054 df = 6 P = 0.983
Av. Peak negative strain rate ≥ -1.33 (s^-1^)	21.7	2.7-173.9	<0.001	21.5	2.5-186.1	0.005	7.7	
Av. Peak-to-peak strain rate ≤ 2.02 (s^-1^)	12.1	3.5-42.3	<0.001	-	-	-	-	
SD time-to-peak positive strain ≥ 101.3 ms	3.6	1.1-11.6	0.026	3.8	0.9-15.1	0.062	3.5	
Constant				0.01	-	0.002	16.2	

Legend: BMI – body mass index; LAV – left atrial volume; Av. Average; SD – standard deviation.

### Predictors of LA stasis

A moderate positive correlation was found between peak positive strain rate and maximum emptying velocity (r = 0.589; P < 0.001) and peak positive strain rate and maximum filling velocity of the LAA (r = 0.651; p < 0.001). Peak negative strain rate was also found to be associated both with maximum emptying velocity (r = -0.513; p < 0.001) and maximum filling velocity of the LAA (r = -0.552; p < 0.001). No significant correlation was observed between peak negative strain and LAA flow velocities and only a trend for a very slight association was observed between peak positive strain and both LAA maximum emptying velocity (r = 0.231; p = 0.066) and LAA maximum filling velocity (r = 0.222; p = 0.078).

On univariate analysis, body mass index, AF episode duration, indexed left atrial volume, peak positive strain rate, peak negative strain rate, peak-to-peak strain rate and time to peak positive strain were predictors of thrombus or sludge on transesophageal echocardiogram. However, only AF duration, peak negative strain rate and time-to-peak positive strain remained significant on multivariate analysis (Table 3). The area under the curve for the estimated probabilities using the obtained logistic regression model was 0.89 (95%CI 0.81-0.96; P < 0.001). The same logistic regression model also displayed a high discriminative capability for the prediction of dense spontaneous echocardiographic contrast: c-statistic = 0.81; 95%CI 0.71-0.91; P < 0.001).

## Discussion

Our data suggest that left atrial mechanical dysfunction, as assessed through peak negative longitudinal left atrial strain rate and time-to-peak positive strain, are associated with the presence of LAA thrombus or sludge. These findings are important not only due to their possible application in the selection of patients for transesophageal echocardiogram before catheter ablation or cardioversion of AF, but also for risk stratification of AF. These parameters may relate to prognosis and clinical endpoints, since LAA thrombus [12,13], sludge [11,14], spontaneous echocardiographic contrast [12,15,16] and low flow velocities in the LAA [11,12] have been associated with a worse outcome in patients with AF.

The described association may be explained by the following: higher left atrial dyssynchrony and lower deformation (compromised contraction and relaxation) may predispose to stasis (as shown by the correlation with flow velocities in the LAA) and subsequently to increasing severity of spontaneous echocardiographic contrast, sludge and thrombus formation. Furthermore, it has been suggested that left atrial wall fibrosis, assessed through delayed enhancement magnetic resonance imaging is inversely related to the strain and strain rate derived from vector velocity imaging echocardiography [17]. Therefore, alongside with fibrosis, loss of left atrial endocardium integrity may occur, which may partially account for the increased pro-thrombotic profile in the atrial milieu in patients with compromised left atrial mechanical function.

The average strain and strain rate values in our population of patients with AF may be considered low compared to the standard values proposed by Cameli et al. [18,19]. However, despite the different methodology, they are in the range of what has been found in previous descriptions of strain and strain rate in patients with AF [19,20].

In our sample, left atrial size (indexed left atrial volume) was not an independent predictor of left atrial stasis, after adjustment to other echocardiographic parameters and AF episode duration. This may mean that previously described associations between atrial size [21,22] and transesophageal echocardiogram changes may be due to the mechanical function alterations that occur in the dilated atrium, rather than to atrial size alone. Furthermore, this may also explain why, unlike left ventricle ejection fraction, left atrial size has failed to consistently associate with thromboembolism in non-valvular AF [23] and is not used alongside other validated clinical variables in risk stratification [24].

Previous investigations support the assessment of left atrial strain and strain rate in patients with AF, with promising results [17,25,26] concerning its association with fibrosis and thromboembolism, which suggest a possible role as a predictor of poorer cardiovascular outcomes and risk of stroke. A case–control study has found that patients with permanent AF and a history of stroke have lower peak positive longitudinal left atrial strain during atrial filling and peak strain rate in the reservoir phase (measured through speckle tracking) when compared to matched controls with no history of stroke [26]. In a cross-sectional study of patients with AF, global longitudinal left atrial strain (assessed through speckle tracking) was lower in patients with a higher thromboembolic risk (CHADS_2_ score ≥ 2) [27]. A recent case–control study composed of patients with AF and a low-risk CHADS_2_ score (≤ 1 assessed prior to cerebrovascular events) suggested that reduced peak negative left atrial strain might identify those at risk for stroke [25].

Besides this preliminary clinical evidence, data concerning a possible association between left atrial strain and strain rate with left atrial stasis are scarce. A study using tissue Doppler imaging has shown a moderately strong positive association (r = 0.73; p = 0.007) between mean left atrial systolic strain and LAA appendage emptying velocities [28], contrary to what we found in our sample. Leong et al. have found that speckle tracking derived parameters are more rapidly measured and more accurate than tissue Doppler for the discrimination of the presence of moderate-severe left atrial spontaneous echocardiographic contrast [29]. Patients with AF are also known to present higher degrees of left atrial dyssynchrony [30]. However, to the best of our knowledge, its association with the presence of left atrial stasis had not yet been demonstrated.

Unlike previous studies, our investigation includes only patients that were assessed during an AF episode. Using speckle tracking for the quantification of left atrial deformation and dyssynchrony we have provided the first evidence towards the association of these novel parameters with the presence of LAA thrombus or sludge. However, one point of our investigation must be highlighted: due to its cross-sectional design, we can only conclude towards an association of compromised left atrial deformation with left atrial stasis. Therefore, since no follow-up was performed, no causal association between left atrial dysfunction and thrombus or sludge formation can be inferred.

Most data of a possible association between compromised left atrial deformation and a pro-thrombotic state in AF are based on strain. Some reasons may explain why in our sample the association was found for strain rate: Unlike other studies where most patients were assessed in sinus rhythm (e.g. ±60% in Azemi et al. [25]), all patients in our study were in AF at the time of the exam. Also, our left atrial strain rate was slightly higher than in other studies (i.e. Saha et al. [27]), which may probably be due to the different method used for its measurement (peak positive and peak negative strain rate in a fibrillating left atrium), rather than peak systolic or end systolic strain rate, which may be difficult to ascertain when no systole or diastole can be clearly identified. From a pathophysiologic point of view, there may be a rationale for an association of left atrial strain rate rather than strain with atrial stasis, since probably the speed at which deformation occurs may be more strongly related to stasis than the overall amount of deformation. Additionally, left atrial strain is included as a predictor, as far as time to peak positive strain (traducing atrial dyssynchrony) is concerned. Lastly, the presence of a small sample with only 16 patients with left atrial appendage thrombus or sludge may lack the sufficient statistical power for revealing an association with left atrial strain, mainly since the degree of the association seemed to be smaller than what was verified for strain rate.

### Limitations

Comparison of groups with a discrepancy in size may be associated with some issues. First, a small group of patients may be more prone to being affected by the presence of outliers and therefore, the average value of some variables may not be truly representative. However, in this sample of patients, standard deviations of the assessed echocardiographic variables were smaller in the group of patients with thrombi or sludge, which render the chance of selection bias or outliers less likely. Second, small samples may sometimes lack statistical power to demonstrate the presence of smaller significant statistical differences. For example, patients with thrombus or sludge display lower absolute values of left atrial strain, which fail to achieve statistical significance possibly due to insufficient power of the sample.

Our data were obtained using software that was designed for the assessment of the left ventricle, but still allowed the automatic definition of a region of interest and tracking of speckles in the left atrium. We do not know if using dedicated software to the left atrium would have changed our observations. However, we think it would likely improve the quality of tracking (namely in S_6_) and the total percentage of patients where tracking in the 6 apical 4-chamber segments was achieved.

Due to deficient endocardial border definition of the left atrium in 2-chamber view in a significant number of patients, we have chosen not to evaluate these segments. We acknowledge that having a global evaluation of the left atrium, ideally with 3D echocardiography, may provide more accurate and precise data. However, this could lead to a more complex and time-consuming procedure.

## Conclusions

Left atrial mechanical dysfunction assessed through speckle tracking seems to be associated with a higher prevalence of the different markers of left atrial stasis. These findings suggest a possible application of this technique in the selection of patients with AF for transesophageal echocardiogram. Moreover, there may be a rational for assessing left atrial deformation in the context of risk stratification of AF, but this should be further explored in future trials.

## Abbreviations

AF: Atrial fibrillation; LA: Left atrial; LAA: Left atrial appendage; E: Early diastolic filling velocity; E’: Early diastolic tissue velocity.

## Competing interests

The authors declare that they have no competing interests.

## Authors’ contributions

RP designed the study, performed the analysis of data along side with AF and wrote the first draft of the manuscript. MJF, LG and SBo discussed and revised the study design. Data was acquired by AF, JT and AB. All authors actively revised the first draft of the paper, providing suggestions and corrections. Input from reviewers was used for preparing a new version of the paper, which was prepared and revised by all authors. SBa provided all the necessary English language support for the preparation of the final version. All authors read, revised, accepted the final version of the manuscript and confirm the accuracy or integrity of data.
